# Dr. Spark on Spasm of the Uterus

**Published:** 1813-10

**Authors:** William Spark

**Affiliations:** Ipswich


*To the Editors of the Medical and Physical Journal.*

GENTLEMEN,

YOUR correspondent Obstetricus has expressed a wish that I would state clearly the distinguishing symptoms between those tedious cases of labour arising from spasmodic affection of the uterus, and those arising from rigid muscular fibre. I thought this had been done when I stated that "the patients most subject to spasm were weakly, or what are said to be nervous. The general symptoms, a frequent desire to void urine in small quantities; the os uteri little dilated, and during pain instead of dilating it contracts; and the spirits of the women are depressed." The laborious cases arising from rigid muscular fibre attend strong young women, or those advanced in years with first children. To the symptoms of spasm I can add one more, viz. the child is in frequent motion, or, as the mother describes it, it worries.

I hope from this description every practitioner will be able to discriminate between the two cases. Obstetricus has mistaken my meaning when I said that I supposed opium would have no effect in cases arising from rigid muscular fibre: my idea was confined to the relief of the case.

I am, Gentlemen,

Your obedient Servant,

*Ipswich.* WILLIAM SPARK, M.D.

P.S.—Should Obstetricus have another case of puerperal convulsions, will not opium *save* his patient better than brandy? I suppose those cases partake much of spasm.

